# Challenges and opportunities in setting up a phase III vaccine clinical trial in resource limited settings: Experience from Nepal

**DOI:** 10.1080/21645515.2020.1855955

**Published:** 2021-02-01

**Authors:** Tarun Saluja, Bishnu Rath Giri, Shipra Chaudhary, Dipesh Tamrakar, Piush Kanodia, Sonali Palkar, Sridhar Vemula, Suchada Chinaworapong, Bomi Kim, Birendra Prasad Gupta, Sue Kyoung Jo, Sanet Aspinall, Ganesh Kumar Rai, Duncan Steele, Jerome H. Kim, T. Anh Wartel, Sushant Sahastrabuddhe

**Affiliations:** a International Vaccine Institute, Seoul, Republic of Korea; b Kanti Children’s Hospital, Kathmandu, Nepal; c B P Koirala Institute of Health Sciences, Dharan, Nepal; d Kathmandu University School of Medical Sciences, Dhulikhel, Nepal; e Nepalgunj Medical College, Nepalgunj, Nepal; f Bharti Hospital, Pune, India; g Ardent Consulting (Pty) Ltd, South Africa; h Bill & Melinda Gates Foundation, Seattle, USA

**Keywords:** Capacity building, resource-limited settings, phase III, vaccine trial, clinical trial

## Abstract

Clinical trials are complicated, time-consuming and costly. From the initial screening, informed consent and recruitment of the participants’ to study completion, the sponsor must undertake a wide array of complex and closely monitored operations, complying with international standards for human subject research and local requirements. Conducting these studies in an underdeveloped country, with limited resources, infrastructure, and experience with regulated clinical trials adds to this complexity. The initial site selection, set up and preparatory activities for the clinical trial are crucial to minimizing the risks to both participants and to successful completion during the subsequent study execution.

In this paper, we describe the experience and lessons learned of building clinical trial site capacity in terms of infrastructure and human resource development for a Phase III vaccine clinical trial. We believe that sharing the experience of setting up a clinical trial in a resource-limited country will enable other entities contemplating clinical research in these countries, to prepare and plan ahead, to minimize the impact of barriers, and to contribute to bringing more studies to the countries where people live with the burden of vaccine-preventable, poverty-associated diseases.

## 1. Introduction

Clinical trials are any investigation in human participants intended to discover or verify the clinical, pharmacological, and/or other pharmacodynamic effects of an investigational product.^1^ Research studies on human participants are designed to answer specific questions about biomedical interventions, including new treatments such as novel vaccines, drugs, dietary choices, dietary supplements, medical devices, and other interventions.^2^ Individual clinical trial results in one population or age group may not be applied universally because countries vary in disease burden, genetic structure, immune responses, population dynamics, culture, and perceptions, and what is appropriate in one place might not be in another.^3^

Some interventions shown to be efficacious in high-income countries are not similarly effective when used in other populations in low and middle-income settings.^4,5^ For example; a Cochrane review of rotavirus vaccines in young healthy children, based on 11 randomized-controlled trials (RCTs) of Rotarix® and six RCTs of RotaTeq®, showed protection against severe rotavirus gastroenteritis after 1 and/or 2 years of follow up, ranging from approximately 80–90% with modest waning over the period of observation in high resource settings as compared to approximately 40–60% efficacy over 2 years of follow up in low-resource settings.^6^ Regional/Country and population-specific local data helps global policy makers, such as the World Health Organization and Gavi, The Vaccine Alliance as well as national governments in making evidence-based decisions and allocation of their resources.

Conducting clinical trials is a complicated, time-consuming and costly task regardless of the region. From the initial screening, informed consent and recruitment of the participants’ to study completion, the sponsor must undertake a wide array of complex and closely monitored operations, complying with international standards for human subject research and local requirements. Conducting these studies in an underdeveloped country, with limited resources and infrastructure, and limited experience with regulated clinical trials adds to this complexity. Despite the burden of infectious diseases, there may not be sufficiently trained and experienced competent sites and personnel capable of executing the clinical trials needed to address these problems. Consequently, these countries are under-represented in research due to lack of commercial viability and research capacity.^7,8^

Barriers to conducting clinical trials vary widely between countries and normally are not considered in the early planning phase by sponsors.^9^ There is little known about the conduct and quality of research in countries that have relatively little clinical research experience.^10–13^ With more understanding of these settings, there is also a growing realization that many countries in the developing world are not using the enormous research potential offered by their health care services.^14^ If enhancing clinical trials in developing countries is being considered, then identifying barriers and designing context-appropriate strategies are critical for success.

The International Vaccine Institute (IVI), based in Seoul, Republic of Korea has been involved since its inception in developing vaccines for diseases of the most impoverished populations, such as cholera and typhoid. Various modeling studies have estimated that the typhoid burden ranges from 12 million to 21 million cases per year and 129 000 to 145 000 deaths annually worldwide. The disease burden is high in low- and middle-income countries, particularly in Asia and sub-Saharan Africa.^15–20^ To tap the enormous potential and to cultivate the research culture (developing research capacity), in low-resource settings where typhoid fever burden is high, IVI elected to conduct a phase III clinical trial in Nepal, a low-income country where few late-stage clinical trials are conducted. Capacity building in developing countries is one of the mandates of IVI, and the proposed study was a typhoid conjugate vaccine clinical trial, a disease endemic in the local population.^21,22^

Proposed clinical trial entitled “A phase III multicenter, observer-blinded, randomized, active-controlled, immune non-inferiority and safety study of Diphtheria Toxoid Conjugated Vi-polysaccharide typhoid vaccine compared to Typbar TCV® in healthy 6 months-45 years aged Nepalese participants” was planned with a sample size of 1800 participants divided into 3 age strata, 6 months to less than 2 years, 2 to less than 18 years, and 18 to 45 years, having 600 participants each.^23^

## 2. Preparation for clinical trial

Understanding the complexities of this project, a blueprint with detailed plan was developed 18 months before the proposed trial start. The plan included activities for regulatory understanding, site identification and capacity building, media sensitivity, risk analysis, and mitigation. All existing IVI internal Standard Operating Procedures (SOPs) on clinical trial conduct were part of this plan, and the first major step was to set up a dedicated team/task force for this purpose. This team was given the responsibility of identifying the challenges and preparing the sites, per study requirements, in coordination with internal and external stakeholders.

### 2.1 Regulatory and ethical consultations

The overall aim of the Vi-DT typhoid conjugate vaccine (TCV) development program at IVI is to achieve local licensure of the vaccine in the country of manufacture (South Korea for the vaccine manufactured by SK bioscience) followed by the World Health Organization (WHO) pre-qualification (PQ) which is pivotal to be eligible for purchase by United Nations agencies, like United Nations Children’s Fund (UNICEF), or Gavi, The Vaccine Alliance.^24^ As part of this strategy, we organized multiple consultations with WHO PQ team for PQ requirements, Korean Ministry of Food & Drug Standard (KMFDS) for an export only license due to the lack of local endemic typhoid infection, and the Nepal Health Research Council (NHRC) for clinical trial approval and Department of Drug Administration (DDA), Nepal for import permission and local licensure for use in clinical trial. A series of meetings were held with regulatory authorities, outlines of the project were presented and after getting written approvals from the authorities, project preparation activities were subsequently launched in Nepal.

### 2.2 Site identification

Conducting a clinical trial in Nepal was a challenge due to lack of well-established clinical trial sites and experienced senior principal investigators with the required infrastructure and research staff experienced in conduct of trials according to the International Committee for Harmonization – Good Clinical Practices (ICH E6 (R2)) guidance.^25^ Keeping in mind these limitations, site identification and qualification became the first critical step to ensure smooth study conduct fulfilling the required international quality standards.

Given the large sample size of 1800, it was decided to set up and engage at least 4 sites for participant recruitment. Factors that were considered included, participant recruitment per week (slow due to lack of experience), follow up visit requirements, geographic representation across the country, urban/semi-urban/rural, social mobilization, community engagement, and awareness.

At the time of site assessment and selection, there was one site actively involved in a vaccine clinical trial in the Kathmandu valley, and it could not be utilized due to participation in a different typhoid conjugate vaccine study, with the WHO pre-qualified vaccine.^26^ This required us to look beyond the conventional site in the Kathmandu valley and throughout various regions in Nepal. We started an extensive search, held discussions with relevant stakeholders and sought recommendations from key opinion leaders to develop a first list of potential sites. After developing the list of potential sites, we contacted the potential site investigators and senior leadership to gauge their interest in participating. A non-disclosure agreement was sent to the Investigators before the study was discussed. Based on telephonic discussions and completion of a site feasibility questionnaire, prospective sites were screened to move to the next stage of site selection. After initial screening discussion, we conducted site qualification visits to assess the ability of the staff and infrastructure of clinical research site to conduct the study in a safe, cooperative, and timely manner. The purpose of the site qualification visit was to investigate the operational, managerial, technical, and clinical capabilities of the participating sites, which helped in identification of gaps to be addressed prior to initiation of recruitment and execution of the phase III trial according to required regulatory, ethical and quality standards. Some of the site evaluation criteria from the SOP for site qualification are listed in Table 1.

**Table 1.** Site evaluation criteria

Site’s research interest
Support from Institution’s higher authorities/leadership/management
Available infrastructure (Laboratory/equipment/storage devices/space like area for ICF, medical history, blood draw and vaccination etc.
Availability of qualified staff/human resources
Access to well defined population/community outreach/catchment area
Community image/trust
Past experience with clinical trials/surveillance studies
Geographical feasibility/well connected transport facilities
Communication facilities (Internet/mobile network)
Media sensitivity
Budget requirements
Contract obligations
Regulatory requirements (NRA/Site IRBs)
Training requirements

During the site qualification visit, Project Lead (PL)/designee discussed the study-specific requirements with the potential site staff, checked and documented various evaluation parameters against the site qualification visit checklist. Multiple photographs were taken during the site visits for discussion with the project team back at IVI headquarters.

**Figure 1.**
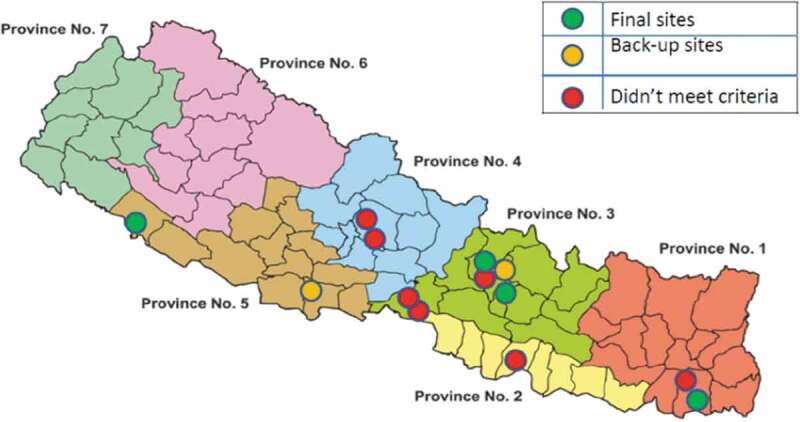
Geographical distribution of trial sites in Nepal

Based on the data gathered, investigators/sites were shortlisted. A detailed comparative site capabilities table highlighting the strengths and weaknesses of the assessed sites was generated and discussed thoroughly within the project team. During this process, we reached out to a total of 20 sites for feasibility and based on the telephonic discussion and site feasibility questionnaire, 13 sites were shortlisted for further assessment (site qualification visit) by IVI staff. Of these 13 sites, 7 sites were initially selected and visited during August 2018 by an external consultant to conduct a thorough gap analysis of site capacities. Finally, four sites were selected for site preparation along with 2 back-up sites Figure 1.

Selected sites’ IRBs were contacted through respective site’s PI and availability of IRB SOPs was confirmed. Wherever needed, efforts were made to strengthen the IRBs by inviting IRB members for various training programs. Site IRBs were encouraged for frequent inspections.

**Figure 2.**
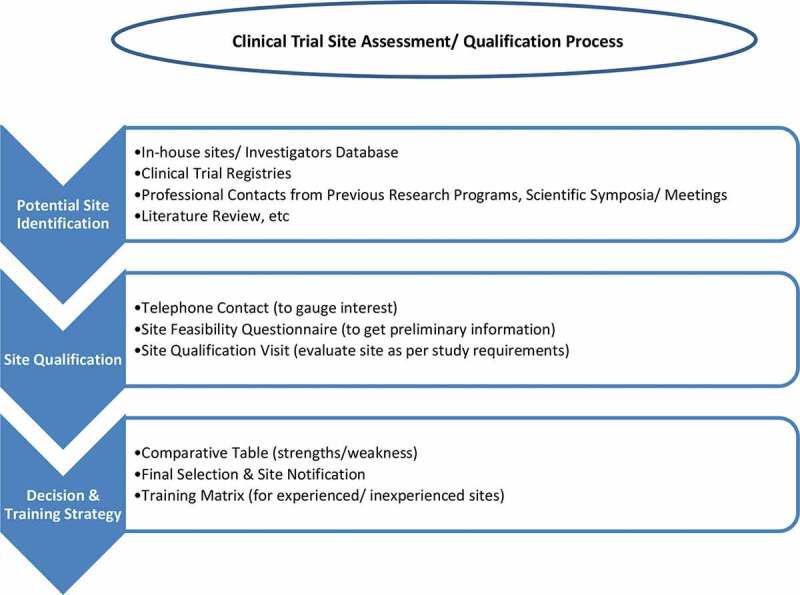
Site assessment flowchart

All the sites and study teams were informed of the final results as per study-specific requirements in writing/e-mails. Entire process of sites’ selection has been summarized in Figure 2.

### 2.3 Clinical site team recruitment

Understanding the site’s capabilities and willingness to participate in this project was the first crucial step. Thereafter, the project team developed and planned the human resource requirement for each site and a basic team plan was shared and discussed with each site. It was understood that clinical trial sites in LMICs, based in Health Care Facilities or Hospitals, are overburdened by the pressing needs of routine patient care; therefore, staff planning was needed to ensure proper time allocation and resources of each member for the research activities without affecting the ongoing patient care. One critical element was the identification and training of young investigators who could take a leadership role in fostering the scientific and research-oriented culture at the site. Site-specific team requirements were discussed and agreed upon by site authorities, including the time allocation required by the potential site study team.

**Figure 3.**
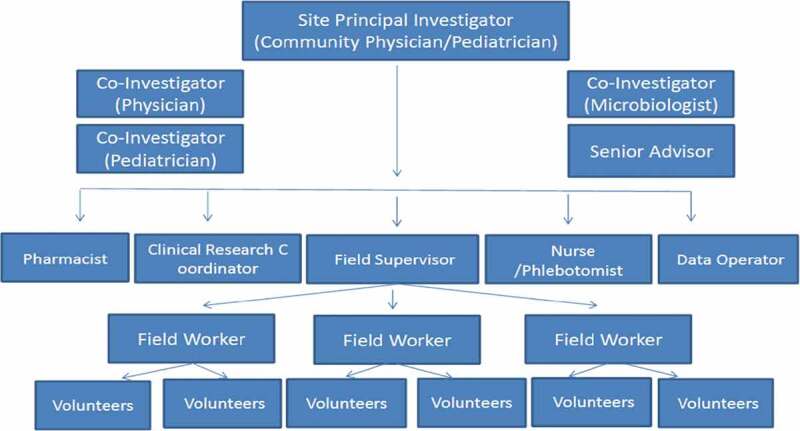
Site staff basic requirement

Note: Back-up of each staff was ensured at each site

A basic staff team Figure 3 was planned at each site; each staff member, except for the site principal investigator, had a designated back-up. A study team consisting of approximately 30 clinical research staff was planned. Each site had at least 2 study coordinators, two study nurses and two medical officers, who were full time dedicated to site research activities. Senior clinicians and academic staff at each site were requested to be Senior Advisors to the project.

### 2.4 Sites’ training strategy

**Figure 4.**
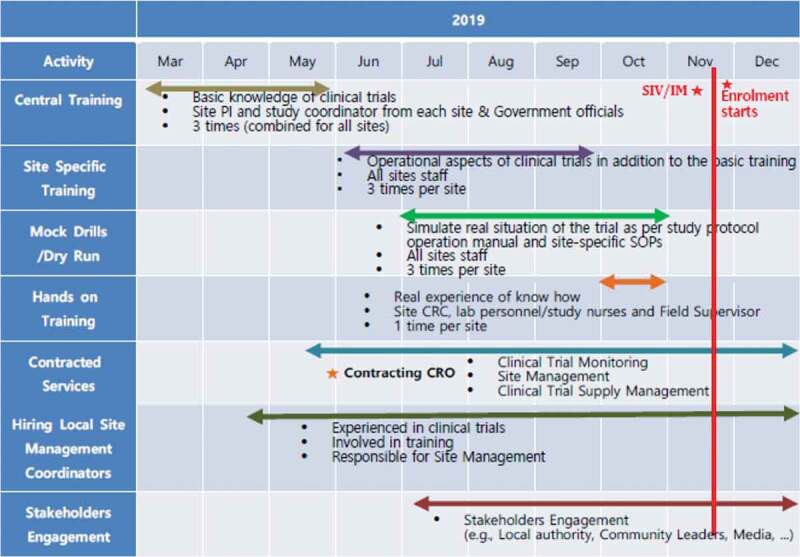
Training matrix & contracted services

For the experienced clinical trial sites, a standard training plan is implemented, starting from Investigator meet trainings, site initiation visit trainings, and on-the-spot training, as needed, during routine monitoring visits. In Nepal, however these potential clinical trial sites had no previous experience, and sites were shortlisted based on evaluation criteria mentioned earlier Table 1. Therefore, an extensive training plan was put in place with several training waves starting with basic knowledge of ICH-GCP to in-depth study-related procedures and activities for ensuring the rights, well-being, and safety of the study participants. The sponsor team believed that the key to scientific success resides in trained human resources and that, emphasis should be on training in an equitable, respectful way and on establishing long-lasting, sustainable partnerships. Therefore, a 8-month, robust training schedule including, central, site-specific, mock drills/dry runs, and hands-on trainings etc. was put in place Figure 4. All the trainings were carried out by IVI along with an independent GCP expert.

#### 2.4.1 Central trainings

The initial ‘central training sessions’ included three key members from each of the sites, i.e. each site principal investigator, co-investigator and co-ordinator who were invited to Kathmandu for training. A total of three central trainings were conducted per site. The purpose of central trainings was to familiarize the staff with the basic terminology used in clinical research and general principles of clinical research and ethics. Topics covered during the trainings were Good Clinical Practices (GCP), Clinical Development Overview, Pre-clinical requirements, First in Human Studies (FIH), Practical Management of Clinical Trials, and important points for beginners in clinical trials, based on the vast experience of IVI conducting clinical trials for 20 years.

The contents of the trainings were adjusted from the first to the third central training, depending on the perceived knowledge level of the participants by means of post-training assessments and feedbacks. The training was made progressively more detailed and study-specific.

#### 2.4.2 Site-specific trainings

Three ‘site-specific trainings’ spaced roughly 1 month apart, were provided at each site, keeping in mind site-specific requirements and the bigger site teams including all Investigators, Nurses, Lab technicians, Data operators, Field supervisors & Volunteers. Training was conducted in English and local languages. The major focus of these trainings was the operational aspects of clinical trials in addition to the basic training on GCP for all staff.

#### 2.4.3 Mock drill/dry run

Mock drills were planned immediately after each site-specific training. The purpose of mock drills was to simulate the real situation of the clinical trial per the study protocol and study operation manual requirements. Role play with a focus on the informed consent process, case report form, electronic data entry, sample preparation, and handling, sample storage, and safety- reporting were performed several times. In addition, extensive training was done over the site-specific SOPs, which were developed in collaboration with an independent consultant agency, and discussed with the study sites before implementation.

#### 2.4.4 Hands on training

In spite of the extensive training – including 3 central, 3 site-specific and 3 mock drills, it was important for the key site staff to observe and learn from an ongoing clinical trial. IVI contacted established clinical trial sites in India with which we were familiar and finally settled on the site at Bharti Hospital, Pune, Maharashtra, India for on-site training and observation. This site has successfully conducted multiple clinical trials and is well known for its quality adherence. IVI worked with the principal trainer and team at Bharti Hospital to prepare a week-long extensive training workshop agenda. We identified two key staff from each Nepalese site, and they were invited for hands-on training at Pune. Since participants were invited to observe an ongoing trial, a Non-Disclosure Agreement (NDA) was signed by all the participants before the start of the training workshop. Site principal trainer informed the ongoing study sponsor of this training exercise and necessary permissions were secured. The purpose of this hands-on training was to give participants on-site observation and experience of conducting a clinical trial, and they were expected to observe and understand various aspects of the ongoing clinical trial, including screening, informed consent, recruitment, study visits, etc.

At the beginning of the workshop a pre – assessment test was conducted for the trainee participants which was based on General Principles of Clinical Research and Ethics. During the workshop, emphasis was given to the ethical conduct of clinical trials, the voluntary nature of trial participation, volunteer safety and well-being, and documentation. The Trainers at Bharti Hospital were requested to conduct a post-training assessment using a questionnaire based on the study protocol. Assessment results were shared with the sponsor for further development of individual trainees.

Activities discussed and observed during the hands-on training are summarized in Table 2.

**Table 2.** Activities observed during hands on training

1.	Informed consenting and assent process
2.	Screening & enrollment process
3.	Blood sample collection
4.	Randomization process
5.	IP administration & accountability
6.	Explaining adverse events captured in the diary card
7.	Serum sample handling, processing & labeling
8.	Development of source documents as per protocol for screened & enrolled participants
9.	Collection of source documents
10.	Data entry from source to EDC(eCRF)
11.	Capture of Adverse events in the source and EDC
12.	Capturing of concomitant medications in the source and EDC
13.	Filling of essential study logs
14.	ISF creation & maintenance
15.	Maintaining of refrigerator, deep freezer and centrifugation process
16.	Reporting of protocol deviations/violations to Ethics committee and sponsor
17.	Sending of updated recruitment trackers to sponsors/CRO
18.	Archival of study documents

### 2.5 Contracted services

#### 2.5.1 Contract research organization

In order to oversee the operational activities during the study conduct with the sponsor, various Contract Research Organizations (CROs) were contacted. Many well-known and global CROs did not participate because they did not have the experience or resources to conduct this kind of study in Nepal. Keeping in mind these challenges, a CRO from India was hired to take care of study monitoring and local project management activities.

#### 2.5.2 Local co-ordinators/consultants

All of the four sites selected were new to the clinical trials. Therefore, in addition to extensive training of the site and the hiring of a CRO for monitoring activities, four additional experts in regulatory activities, laboratory assessment, and clinical trial operational activities were hired: 2 from Nepal and 2 from India who are based in Nepal. This ensured that we had enough support on the ground for crucial capacity-building efforts and management during the study.

### 2.6 Stakeholder engagement/management

As the study recruits participants from the local community, social mobilization and community engagement are critical areas for a successful study, and thus a community engagement plan was put in place. The objectives were, to enhance an informed decision process for parents and children invited to participate in the vaccine trial, to provide accurate and up-to-date information regarding the Vi-DT vaccine and the phase III vaccine clinical trial and to establish a mechanism within the trial site’s health network to detect rumors and/or concerns within the community that might enable the project team to respond accordingly in a timely manner.

**Figure 5.**
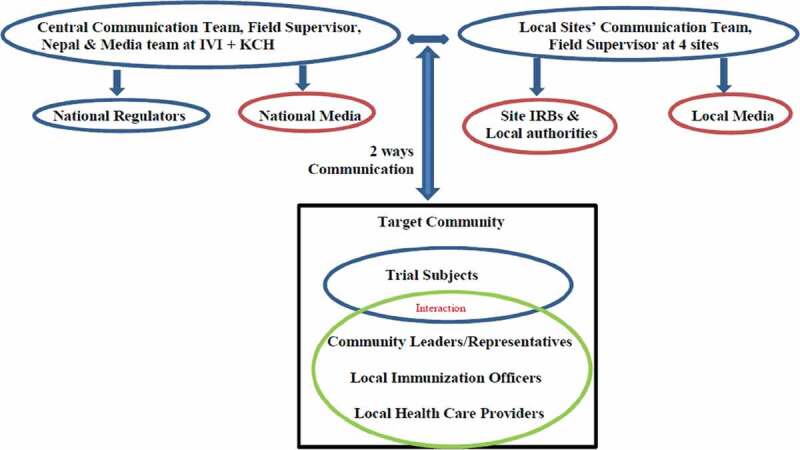
Community engagement & communication plan

To combat common challenges of fear, distrust, and possible preconceived notions about clinical trials or suspicions of ‘research’ in the community, site staff conducted extensive community outreach in their respective site catchment area. Community support for the study was considered essential and activities ranged from staff going door-to-door and visiting local groups to meetings with the district’s public health division, key opinion leaders, locally elected representatives, caregivers, and physicians. A comprehensive dialogue was established with all the stakeholders, explaining the critical aspects of the proposed study and assuring them that the best ethical, medical, and scientific practices would be followed during the study Figure 5. This involved multiple meetings with various groups in the community and ample time for questions and discussion.

### 2.7 Logistics challenges & management

#### 2.7.1 Basic infrastructure

For each clinical trial site, a floor map was developed to help with the efficient use of the space and to fulfil the requirements for a clinical trial site as per ICH-GCP. A minimum of 4 rooms for research activities were set up (i.e., informed consent and counseling room, blood draw & serum separation room, vaccination, and post-vaccination observation room). Separate space was allotted for a waiting area, document storage under lock & key, serum sample storage, vaccine storage, data entry and long-term archive. We also worked with each site to have a proper participants’ flow from entry to exit at each site. Internet with high-speed Wi-Fi with required band width was ensured at each site to take care of electronic data entry during the study conduct.

#### 2.7.2 Equipment/instruments

All the required equipment were purchased locally or from India. Equipment/instruments such as refrigerator for IP storage, deep freezer for serum storage etc. were arranged in duplicate, main & back-up, established at separate places, and were calibrated before the study start and checked routinely per SOP. Calibration certificates were issued & maintained in a separate file for quality checks.

### 2.8 Participant recruitment plan

Recruiting study participants is the most challenging part of any clinical trial and is a major cause of clinical trial delay. To meet the challenge of successful, timely enrollment, site teams were advised to strategize carefully and a study-specific, participant-focused recruitment plan was developed which was specific to each site. A more accurate and realistic assessment of recruitment per week and a true forecasting rate to avoid congestion of study follow-up visits, was determined from various sources of information, such as immunization clinic vaccination records, follow-up OPD visit logs, a well-defined catchment area and available site demographic data. Some sites targeted immunization clinics and follow-up OPDs for participants’ identification with field as back-up, while others targeted field area for participants’ identification as main strategy. Similarly, participants’ retention plan was put in place to ensure minimum dropout, one of the essential component of the plan was frequent contacts with participants by community/field health workers until the study completion.

### 2.9 Study monitoring/quality check plan

To ensure that the study was conducted according to GCP and bearing in mind the relative lack of experience of the sites, we decided to have the first monitoring visit after 2–3 participants were recruited at each site. The purpose of this early monitoring check was to identify any deviations at an early stage, make timely corrections, and retrain appropriately if required. Study monitoring activities were conducted by CRO, while co-monitoring activities were performed by the sponsor clinical operations group, and to ensure quality, regular quality checks were assigned to senior clinical research officials at IVI. In addition, IVI hired an independent agency to ensure quality check evaluations at predefined time points. The reports from these evaluations were shared with IVI and required corrective and preventive actions were put in place accordingly.

### 2.10 Site readiness checks

Before starting the clinical trial, final site readiness check was performed by an independent consultant from South Africa. The objectives of the site readiness check were; 1) To determine whether all regulatory, central, and local ethics approvals and an executed clinical trial agreement were in place; 2) To determine whether minimum staff requirements were in place to fulfil the relevant roles in the conduct of the trial; 3) To determine whether staff has been adequately trained in GCP and in other aspects of trial conduct; 4) To determine whether there were adequate facilities at site to conduct a large vaccine trial; 5) To determine whether back-up power was in place and temperature of vaccine refrigerators and laboratory freezers were monitored and stable; 6) To determine if the medical emergency trolley was in place and available for use at any time; 7) To determine whether the site staff had good understanding of the participant recruitment and enrollment plan; 8)To determine if the site has final standard operating procedures (SOPs) in place like general operations, obtaining informed consent, medical emergency power failure and natural disaster, quality management etc. and had been trained on those SOPs.

During the visit, the following details were checked and verified: organogram of site staff, training records, CVs and job descriptions, investigator site file for all approvals, facility tour of the site, medical emergency trolley, SOPs and study manual of procedure (MOP). Interview with principal investigator and co-investigator was conducted to review and understand the roles and responsibilities of site staff, participant-focused recruitment plan, and participant flow within the site. Furthermore, study staff including physicians and nurses were asked questions around the study procedures on an ad hoc and unplanned manner to ascertain full comprehension of the study protocol.

A detailed report was prepared by the consultant and was submitted to the sponsor which served as a final clearance before enrollment.

## 3. Discussion

In 2015, Li et al. reported the findings of their review of the distribution of global clinical trials and concluded that despite an overall increase in clinical trials in developing countries over the last two decades, progress (number of clinical trials being conducted) made was particularly slow and challenging.^27^ The Global Forum for Health Research report emphasized that strengthening research capacity in developing countries is one of the most effective and sustainable ways of advancing health and development in these countries, as well as helping correct the gap in health research.^28^ Conducting more clinical trials in low and middle-income countries (LMIC) will build confidence both in the sites and among sponsors. The capacity building will strengthen institutions and investigators in LMIC; ultimately, these will impact the level and quality of health care provided in these settings.^29^ In addition, research generated in LMICs help respective governments in policy formulation and implementation of decisions using relevant analyses based upon local data.

One of the missions of IVI is to build the research capacity in developing countries, and the Vi-DT phase III clinical trial provided a crucial opportunity to implement that. Several LMICs were evaluated and Nepal was chosen in close consultation with the Bill and Melinda Gates Foundation (BMGF), Seattle, USA and the vaccine manufacturer, SK bioscience, Republic of Korea. The trial was initiated in November 2019 and is ongoing, however, the eruption of COVID-19 globally has resulted in unexpected delays and new challenges, as the trial activities were put on hold during the pandemic. The close collaboration and support of BMGF, manufacturer, site staff, and various government agencies was critical to the progress till date. The typhoid conjugate vaccine Vi-DT should complete phase III in 2020, and regulatory approval and licensure is expected in 2021. Successful examples of the optimal utilization of Product Development Partners (PDPs) such as IVI for Cholera and Typhoid and PATH for Rotavirus vaccine development programs, highlights the importance of PDPs even for manufacturer-sponsored trials in setting-up and executing clinical trials in resource-limited settings.

Being new to clinical trials, sites had many weaknesses initially that were addressed through extensive planning, preparation, and training. All the sites were eager to learn and adapt, and this facilitated the site set-up activities. The institutional ethical committees at the sites were at various levels of experience, and the strengthening of site ethical review could contribute further to the enhancement of site research capacity.

While preparing clinical trial sites in Nepal, a substantial amount of time; 18 months to be precise, were spent from site identification to eventual readiness for the Vi-DT phase III clinical trial. During the course of site preparation, the sponsor team faced many logistical challenges, for example, difficulty purchasing and calibrating good quality refrigerators, deep freezers and centrifuges. Transportation was another major challenge, due to unpredictable weather conditions leading to local flights delays or cancellations; flight delays varied from 1 to 8 hours depending upon route and season of the year. Absence of a rail network and poor road infrastructure also contributed to the issue. To ensure the smooth execution of the trial, air and road routes were well defined with back-up transport strategies in case of delay or cancellation of flights. For example, prior arrangements were made with World Courier services to supply cold chain boxes capable of maintain required temperatures up to 7 days for serum sample and Investigational Product (IP) transports. Similarly to ensure safety of the IP at sites, 3 sets of IPs, main set, back-up and back-up of back-up set was maintained at 3 different locations.

More clinical research should be done in LMICs and sharing the experience of setting up a clinical trial in a resource-limited country will enable entities contemplating clinical research in these countries to prepare and plan, minimizing the impact of barriers to the conduct of GCP trials in under-resourced settings.
